# Development of highly efficient whole-cell catalysts of *cis*-epoxysuccinic acid hydrolase by surface display

**DOI:** 10.1186/s40643-022-00584-6

**Published:** 2022-08-29

**Authors:** Rui Zhou, Sheng Dong, Yingang Feng, Qiu Cui, Jinsong Xuan

**Affiliations:** 1 Department of Bioscience and Bioengineering, School of Chemistry and Biological Engineering, University of Science and Technology Beijing, 30 Xueyuan Road, Beijing, 100083 China; 2 CAS Key Laboratory of Biofuels, Qingdao Institute of Bioenergy and Bioprocess Technology, Chinese Academy of Sciences, 189 Songling Road, Qingdao, 266101 China; 3 Shandong Provincial Key Laboratory of Synthetic Biology, Qingdao Institute of Bioenergy and Bioprocess Technology, Chinese Academy of Sciences, 189 Songling Road, Qingdao, 266101 China; 4 Shandong Engineering Laboratory of Single Cell Oil, Qingdao Institute of Bioenergy and Bioprocess Technology, Chinese Academy of Sciences, 189 Songling Road, Qingdao, 266101 China; 5 Shandong Energy Institute, 189 Songling Road, Qingdao, 266101 Shandong China

**Keywords:** Whole-cell biocatalyst, *cis*-Epoxysuccinic acid hydrolase, Surface display

## Abstract

Bacterial *cis*-epoxysuccinic acid hydrolases (CESHs) are intracellular enzymes used in the industrial production of enantiomeric tartaric acids. The enzymes are mainly used as whole-cell catalysts because of the low stability of purified CESHs. However, the low cell permeability is the major drawback of the whole-cell catalyst. To overcome this problem, we developed whole-cell catalysts using various surface display systems for CESH[L] which produces L(+)-tartaric acid. Considering that the display efficiency depends on both the carrier and the passenger, we screened five different anchoring motifs in *Escherichia coli*. Display efficiencies are significantly different among these five systems and the InaPbN-CESH[L] system has the highest whole-cell enzymatic activity. Conditions for InaPbN-CESH[L] production were optimized and a maturation step was discovered which can increase the whole-cell activity several times. After optimization, the total activity of the InaPbN-CESH[L] surface display system is higher than the total lysate activity of an intracellular CESH[L] overexpression system, indicating a very high CESH[L] display level. Furthermore, the whole-cell InaPbN-CESH[L] biocatalyst exhibited good storage stability at 4 °C and considerable reusability. Thereby, an efficient whole-cell CESH[L] biocatalyst was developed in this study, which solves the cell permeability problem and provides a valuable system for industrial L(+)-tartaric acid production.

**Graphical Abstract:**

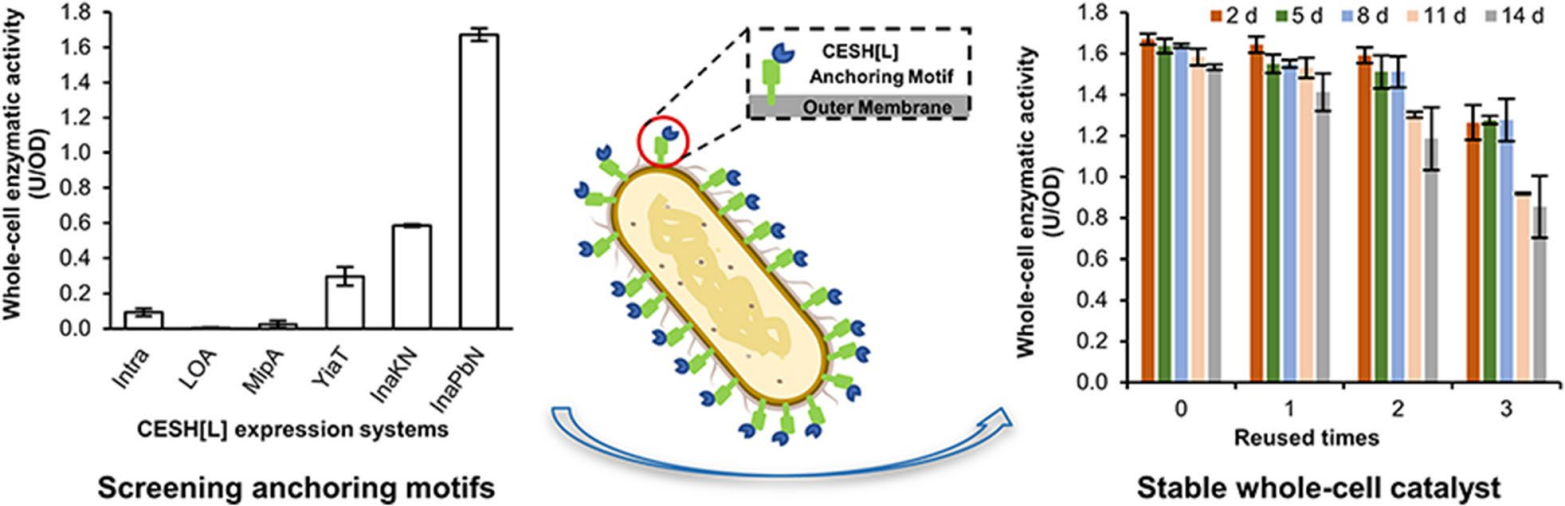

## Introduction

Enantiomeric tartaric acids (TA) are well-known chiral chemical building blocks with broad industrial and scientific applications (Liao et al. 2015; Ling et al. 2021). Bacterial *cis*-epoxysuccinic acid hydrolases (CESHs) can catalyze the asymmetric hydrolysis of *cis*-epoxysuccinate (CES) to form an enantiomerically pure tartrate (Cui et al. 2012; Dong et al. 2018a; Li et al. 2010; Pan et al. 2010). Many bacteria with CESH activity have been used as whole-cell biocatalysts for the industrial synthesis of enantiomerically pure TA for decades (Xuan and Feng 2019). Since the discovery of CESHs, it was found that the CESH producing L(+)-TA (CESH[L]) has low stability and a lot of efforts have been made to enhance the enzyme and whole-cell stability such as whole-cell immobilization (Bučko et al. 2005; Lan et al. 2014; Rosenberg et al. 1999; Sun et al. 1995; Wang et al. 2014; Zhang and Qian 2000), enzyme immobilization (Wang et al. 2017, 2012), and protein engineering (Qiao et al. 2015; Zhang et al. 2016). Furthermore, CESHs are intracellular enzymes and the activity of whole-cell catalysts depends on the cell permeability (Xuan and Feng 2019). Treatments with surfactants or ultrasound have been used to enhance the cell permeability and improve the bioconversion efficiency more or less (Dong et al. 2018b; Jin and Zhang 2003; Rosenberg et al. 1999; Zhang and Qian 2000). The other promising ways to overcome the problem of the low cell permeability include secretion or cell surface display of CESH[L] (Xuan and Feng 2019), but these ways have not been explored as far as we know.

Cell surface display is to attach target proteins on the cell surface by recombinant technologies and is an effective solution to avoid substance transportation, improve biocatalyst’s efficiency, enhance the enzyme stability, and simplify the enzyme preparation process (Baek et al. 2010; Han et al. 2018). This technique has been widely applied in various biotechnical and biomedical areas such as drug screening, biocatalysts, library screening, and biosensors (Pham and Polakovic 2020). Microbial surface display technology offers the advantages of high yield, high productivity, capability to display large-sized targets, and easier process of screening (Park 2020; Pham and Polakovic 2020; Wang et al. 2021). *Escherichia coli* has emerged as one of the most widely used hosts because of its well-studied genome, its well-established genetic toolbox, its high transformation efficiency, and its good compatibility with heterogenous proteins. Regarding surface display systems for *E. coli*, the Lpp-OmpA (LOA) system was the first and the widely used carrier protein (Georgiou et al. 1996). Since then, many candidate proteins, such as MipA (Han 2020) and YiaT (Han and Lee 2015) motifs, located on *E. coli* outer membrane were developed and proved to be efficient for appropriately displaying proteins with various sizes and characteristics. Ice nucleation protein (INP) is also a kind of outer membrane-anchoring protein present in ice nucleation bacteria and various length truncated INP forms were chosen to display proteins (Niu et al. 2015; Wee et al. 2019; Zhang et al. 2019). The success and efficiency of surface displaying are highly dependent on the appropriate choice of an anchoring motif and the properties of the target protein (Lee et al. 2003; Schuurmann et al. 2014).

In the present study, we explored the possibility of the CESH[L] surface display system to overcome the cell permeability problem. To establish a highly efficient system, we screened five well-known anchoring motifs and constructed a series of *E. coli* cell surface display systems for CESH[L]. The efficiency of the surface displays and the whole-cell enzymatic activities were compared and a highly efficient display system for CESH[L] was obtained using an N-terminal truncated INP. The system was further optimized to a very high display efficiency and level and its activity was compared with the soluble CESH[L] in an intracellular expression system. Additionally, the stability and reusability of the CESH[L] display system were also assessed for convenient industrial applications.

## Materials and methods

### Bacterial strains, plasmids, gene sources, and reagents

*Escherichia coli* strains TOP10 and BL21(DE3) were used as hosts for gene manipulation and protein expression, respectively. Plasmids pET28a and pET22b were used for the construction of expression vectors for fusion proteins. All plasmids used in this study are shown in Table 1.

**Table 1:** Plasmids used in this study

Plasmids	Descriptions	Source
pET28a	pBR232 origin, *lacI* coding sequence, Km^r^, P_T7_	Novagen
pET22b	pBR232 origin, *lacI* coding sequence, Ap^r^, P_T7_	Novagen
pET28a-CESH[L]	pET28a derivative, Km^r^, intracellular CESH[L] expression with N-terminal His_6_-tag	(Cui et al. 2012)
pET28a-INPN-SC3	pET28a derivative, Km^r^, source of InaKN gene	Gift from Prof. Chengzhi He
pET28a-InaPbN	pET28a derivative, Km^r^, source of InaPbN gene	(Song et al. 2015)
pET22b-LOA-CESH[L]	pET22b derivative, P_T7_, Ap^r^, fusion expression of LOA-CESH[L]	This study
pET22b-MipAV_140_-CESH[L]	pET22b derivative, P_T7_, Ap^r^, fusion expression of MipAV_140_-CESH[L]	This study
pET22b-YiaTR_232_-CESH[L]	pET22b derivative, P_T7_, Ap^r^, fusion expression of YiaTR_232_-CESH[L]	This study
pET22b-InaKN-CESH[L]	pET22b derivative, P_T7_, Ap^r^, fusion expression of InaKN-CESH[L]	This study
pET28a-InaPbN-CESH[L]	pET28a derivative, P_T7_, Km^r^, fusion expression of InaPbN-CESH[L]	This study

Anchoring motifs in bacterial cell surface display systems used in this study are shown in Table 2. All of these anchoring motifs originate from out membrane proteins in Gram-negative bacteria. LOA is a kind of chimera comprising nine N-terminal residues of lipoprotein (Lpp) and the residues 46–161 of OmpA. The other four kinds of anchoring units are N-terminal truncated forms of four out membrane proteins.

**Table 2:** Anchoring motifs used in this study

Anchoring motifs	Source species	GenBank numbers	Truncated forms
LOA	*E. coli* JM109	AAC74747.1 (Lpp) and AAC74043.1 (OmpA)	124 residues (nine N-terminal residues of Lpp and residues 46–161 of OmpA)
MipAV_140_	*E. coli* BL21(DE3)	AAC74852.1	N-terminal residues 1–140 of MipA
YiaTR_232_	*E. coli* JM109	AAC76608.1	N-terminal residues 1–232 of YiaT
InaKN	*Pseudomonas syringae*	AAB66891.1	N-terminal residues 1–215
InaPbN	*Pseudomonas borealis*	ACB59244.1	N-terminal residues 1–165

Bacterial Genomic DNA extraction Kit, Plasmid Purification Kit, Gel Purification Kit, 2 × Taq Plus PCR MasterMix, and DNA marker were purchased from TIANGEN Biotech (Beijing, China). SDS-PAGE Gel Kit, protein molecular weight markers, and all other analytical grade chemicals were obtained from Sangon Biotech (Shanghai, China). All restriction endonucleases, T4 DNA ligase were provided by Thermo Fisher Scientific (USA).

### Surface display system construction and protein expression

The primers used in this study are listed in Table 3. The primers were synthesized by Sangon Biotech (Shanghai, China). Polymerase chain reactions were performed with a PCR Thermal Cycler (FTC-3000P, Funglyn Biotech, Canada) using 2 × Taq Plus PCR MasterMix (KT205). DNA sequencing was carried out by Sangon Biotech (Shanghai, China).

**Table 3:** Primers used in this study

Primers	Sequence (5′–3′)
LOA-*Nde*I-F	GGAATTCCATATGAAAGCTACTAAACTGGTACTGGGCAACCCGTATGTTGGCTTTGAAATGG
LOA-*Nco*I-R	CATGCCATGGCGCCGTTGTCCGGACGAGTGCC
mipA-F/*Nde*I	GGAATTCCATATGACCAAACTCAAACTTCTGGC
mipAV140-R/*Nco*I	CATGCCATGGCGACGATGCCGTTGCTG
YiaTR232-F/*Nde*I	GGAATTCCATATGTTAATTAATCGCAATATTGTG
YiaTR232-R/*Nco*I	CATGCCATGGCACGATCAATCATCGGGCTGTCGG
InaKN-*Nde*I-F	GGAATTCCATATGACGCTCGACAAGGC
L22b-*Nco*I-F	CATGCATGCCATGGATATGGGCAGCAGCC
L22b-*Xho*I-R	CCGCCGCTCGAGGTCGATACCAGCGG
InaPbN-*Nco*I-F	CATGCCATGGGCATGAACGATGACAAAG
InaPbN-*Bam*HI-R	CGCGGATCCCACCGCTGTCTCCAGCG
L-*Bam*HI-F	CATGGGATCCGGCGGCGGCGGCAGCATGGGCAGCAGCC
L-*Hin*dIII-R	CCAAGCTTGTCGATACCAGCGGTACCACCCAGACGCG

The genomic DNA of *E. coli* BL21(DE3) was isolated and used as the template for PCR amplification to obtain the MipAV_140_ gene by using the primers mipA-F/*Nde*I and mipAV_140_-R/*Nco*I. The genomic DNA of *E. coli* JM109 was served as the template to clone the gene sequences of the anchoring protein fragments LOA and YiaTR_232_ with two pairs of primers LOA-*Nde*I-F and LOA-*Nco*I-R, and YiaTR_232_-F/*Nde*I and YiaTR_232_-R/*Nco*I, respectively. The anchoring motif InaKN gene sequence was amplified from the plasmid pET28a-INPN-SC3 by PCR method with the primers InaKN-*Nde*I-F and InaKN-*Nco*I-R. Each of the four amplified gene fragments above was digested with *Nde*I/*Nco*I, and then ligated to the *Nde*I/*Nco*I sites of the expression vector pET22b, yielding the recombinant plasmids pET22b-LOA, pET22b-MipAV_140_, pET22b-YiaTR_232_, and pET22b-InaKN.

The CESH[L] gene sequence was obtained from the plasmid pET28a-CESH[L] by PCR using the primers L22b-*Nco*I-F and L22b-*Xho*I-R. The resulting CESH[L] full-length gene fragment was digested with *Nco*I/*Xho*I, and then inserted into the *Nco*I/*Xho*I sites of the four previously constructed plasmids (pET22b-LOA, pET22b-MipAV_140_, pET22b-YiaTR_232_, and pET22b-InaKN) to generate the plasmids pET22b-LOA-CESH[L], pET22b-MipAV_140_-CESH[L], pET22b-YiaTR_232_-CESH[L] and pET22b-InaKN-CESH[L] for protein expression.

The plasmid pET28a-InaPbN was used as the template to clone the anchoring unit InaPbN gene sequence by PCR amplification with the primers InaPbN-*Nco*I-F and InaPbN-*Bam*HI-R. The PCR product InaPbN gene fragment was cut by *Nco*I/*Bam*HI and inserted into the plasmid pET28a between the same two restriction sites, obtaining the recombinant plasmid pET28a-InaPbN-*NB*. The DNA sequence of full-length CESH[L] was amplified by using the primers L-*Bam*HI-F and L-*Hin*dIII-R with the template pET28a-CESH[L], digested with *Bam*HI/*Hin*dIII, and cloned into pET28a-InaPbN-*NB* to generate the plasmid pET28a-InaPbN-CESH[L]. All the resultant plasmids were confirmed by DNA sequencing.

The recombinant plasmids were transformed into *E. coli* BL21(DE3) by the CaCl_2_-mediated method. To express the recombinant fusion proteins, overnight cultures of recombinant *E. coli* BL21(DE3) were inoculated at 1:100 and grown in fresh LB broth with appropriate antibiotic (100 μg/ml Amp or 100 μg/ml Kan) at 37 °C to an optical density at 600 nm (OD_600_) of 0.5–0.8. The protein expression was induced with 0.2 mM isopropyl β-D-1-thiogalactopyranoside (IPTG) at 16 °C for 24 h. The IPTG concentration and the expression temperature were further optimized for the InaPbN-CESH[L] expression system. The cells induced with 0.2 mM IPTG at 25 °C for 24 h were used in the following stability and reusability assays for the InaPbN-CESH[L] expression system.

The expression and purification of CESH[L] with the intracellular expression system followed the procedures in the previous study (Cui et al. 2012). The protein concentrations of purified CESH[L] were determined by a BCA Protein Assay Kit (Beyotime Biotechnology, Shanghai, China). A linear curve between the enzymatic activity and the protein amount of purified CESH[L] was established and used to determine the amount of active CESH[L] in the cell lysate and the InaPbN-CESH[L] cell surface display system.

### Trypsin accessibility assay for visualization of display efficiency

To investigate surface exposure of the CESH[L], the proteinase trypsin accessibility test was used. The culture of *E. coli* BL21(DE3) harboring various CESH[L] surface display systems was harvested by centrifugation at 4000 *g* for 5 min at 4 °C. Cell pellets were washed twice with the buffer containing 50 mM Tris–HCl (pH 8.0), then resuspended and standardized to OD_600_ of 5.0 in the same buffer. The intact cells were treated with trypsin with the final concentration of 400 μg/ml for 1 h at 37 °C. The digestion was terminated by adding 2.5 mM PMSF following incubation on ice for 5 min. The *E. coli* BL21(DE3) cells carrying the plasmid pET28a-CESH[L] for intracellular CESH[L] expression were also treated the same way and used as a control. All samples were analyzed by 10% SDS-PAGE gel and stained with Coomassie blue dye.

### Measurement of whole-cell enzymatic activities

The CESH[L] activity of the whole-cell catalyst was measured with a protocol as the previous report with some modifications (Cui et al. 2012): The cell culture was first diluted to OD_600_ of 1.0 with 50 mM Tris–HCl (pH 8.0) buffer. A 200 mM substrate solution was prepared by dissolving epoxy succinate in 50 mM Tris–HCl (pH 8.0) buffer and the pH was adjusted to the desired value with sodium hydroxide. A 200 μl reaction mixture containing 100 μl diluted cell culture and 100 μl substrate solution was incubated at 37 °C for 20 min and then centrifuged at 12,470*g* for 1 min. The supernatant of the reaction mixture was mixed with 80 μl 1 M H_2_SO_4_ and 200 μl 1% ammonium metavanadate. The absorbance at 480 nm was measured by using an EnSpire 2300 multifunction plate reader (PerkinElmer, USA). The concentration of TA was calculated according to the standard curve recorded at various TA concentrations. One unit of enzyme activity was defined as the amount of CESH[L] required to produce 1 μmol L(+)-TA per minute under the assay conditions.

For comparing the surface display system with the cell permeability enhanced intracellular expression system by surfactant treatment, cells expressing intracellular CESH[L] were washed twice and diluted to OD_600_ of 1.0 with 50 mM PBS (pH8.0) buffer. 1.0-ml diluted cell culture was centrifuged at 4000 *g* for 5 min, resuspended in 0.9% sodium chloride solution with 0.2 g/L Triton X-100, and incubated at room temperature for 30 min. Enzymatic activities of 100 μl Triton X-100 treated cell culture and 100 μl supernatant after centrifugation at 12,470 *g* for 1 min were assayed, respectively, with the procedures described above.

### Effects of pH and temperature on the enzymatic activity and stability

The optimal pH of the surface-displayed InaPbN-CESH[L] was determined by measuring the whole-cell enzymatic activities in different buffers with pH ranging from 6.0 to 10.0. The stability of the surface-displayed InaPbN-CESH[L] at different pH was determined by incubating the suspended cells at 4 °C for 7 days and the whole-cell enzymatic activity of the cells was assayed every day. The effects of temperature on surface-displayed InaPbN-CESH[L] were determined by assaying the whole-cell enzymatic activities every day during incubating the suspended cells in 50 mM Tris–HCl (pH 8.0) at different temperatures (4 °C, 16 °C, 25 °C, and 37 °C).

### Re-usability assays of the InaPbN-CESH[L] display system

The InaPbN-CESH[L] expression cells suspended in 50 mM Tris–HCl (pH 8.0) were incubated at 4 °C for different days and the re-usability of the cells was assessed by repetitive enzymatic assays. After the enzymatic reactions with substrates, the cells were centrifuged and the supernatants were used to determine the TA concentration as described in the previous section, while the pellets were washed with 50 mM Tris–HCl (pH 8.0), resuspended in the same buffer, and re-used in the next round enzymatic assay.

### Statistical analysis

All data presented are the averages of three independent biological replicates within each experiment. Statistical analysis was performed using the *t*-test statistical method in Microsoft Excel.

## Results

### Construction of CESH[L] surface display systems on E. coli cells

To obtain an anchoring motif that is suitable for CESH[L] surface display on *E. coli* cells, five anchoring motifs including three endogenous motifs (LOA, MipAV_140_, and YiaTR_232_) and two heterogenous INP truncation motifs (InaKN and InaPbN) were chosen to construct the surface display systems. The gene of each anchoring motif and the gene of CESH[L] with codon-optimization for *E. coli* were sequentially cloned into pET vectors for protein expression. Considering the internal restriction sites, the gene of the anchoring motif for LOA, MipAV_140_, YiaTR_232_, and InaKN was first inserted into the *Nde*I/*Nco*I sites of pET22b, and the gene of CESH[L] was then inserted into the *Nco*I/*Xho*I sites, while the genes of the anchoring motif InaPbN and CESH[L] were inserted into *Nco*I/*Bam*HI sites and *Bam*HI/*Hin*dIII sites of pET28a sequentially. The five recombinant plasmids were obtained and each plasmid was transformed into *E. coli* BL21(DE3), forming five CESH[L] surface display systems. Using these systems, the fusion proteins would be displayed on the cell surface after the protein expression is induced by the addition of IPTG.

### Determination of surface display efficiency of the five display systems

**Fig. 1:**
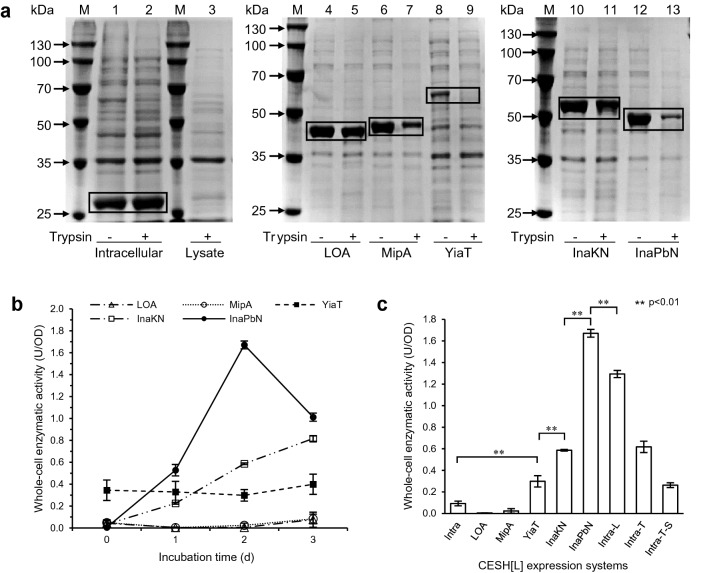
CESH[L] surface display efficiencies of different anchoring motifs. **a** SDS-PAGE analysis of the whole cells or the cell lysate with and without trypsin treatment. Lanes 1–2, whole cells of intracellular CESH[L] expression system without and with trypsin treatment; Lane 3, trypsin-treated cell lysate of the intracellular CESH[L] expression system. Lanes 4–13, each pair of lanes are trypsin-untreated and treated whole cells expressing LOA-CESH[L], MipAV_140_-CESH[L], YiaTR_232_-CESH[L], InaKN-CESH[L], and InaPbN-CESH[L], respectively. Lanes M, standard protein molecular weight markers. **b** Whole-cell activities of various CESH[L] expression systems incubated at 4 °C for different days. **c** Comparison of the surface display systems and intracellular expression system. Intra, LOA, MipA, YiaT, InaKN, and InaPbN represent the cells expressing intracellular CESH[L], LOA-CESH[L], MipAV_140_-CESH[L], YiaTR_232_-CESH[L], InaKN-CESH[L], and InaPbN-CESH[L], respectively. Intra-L, Intra-T, and Intra-T-S represent the whole-cell lysate, the Triton X-100 treated cells, and the supernatant after the Triton X-100 treatment, respectively, of the intracellular expression system. The activities of all surface display systems were measured after 2-day incubation at 4 °C, while the activities of the intracellular expression system were measured without incubation. Symbols ** indicate significant differences with p-values less than 0.01

The surface display efficiency of the five display systems was first assessed by trypsin accessibility assays (Fig. 1a). Trypsin cleaves proteins on the C-terminal side of lysine and arginine residues, so proteins attached to the cell surface with extracellular exposure can be digested by trypsin while the intracellular proteins are inaccessible by trypsin. The trypsin accessibility can be easily determined by SDS-PAGE analysis of trypsin-treated and untreated whole cells, which provides the information of the target protein locations (Maurer et al. 1997). To evaluate whether trypsin accessibility assay is effective for CESH[L], whole cells and cell lysate of the CESH[L] intracellular expression system (Cui et al. 2012) were treated with trypsin. SDS-PAGE analysis showed the same intact CESH[L] bands for the trypsin-untreated and treated cells, but the CESH[L] band of the cell lysate after trypsin treatment almost disappeared (lanes 1–3 in Fig. 1a). This demonstrated that CESH[L] can be digested by trypsin, but trypsin cannot pass through the cell membranes. Therefore, trypsin accessibility assay can be used to ascertain the surface localization of the expressed CESH[L].

SDS-PAGE analysis of trypsin-treated and untreated whole cells expressing fusion proteins LOA-CESH[L], MipAV_140_-CESH[L], YiaTR_232_-CESH[L], InaKN-CESH[L], and InaPbN-CESH[L] showed that the protein expression levels and the cell surface display levels were different in these systems (Fig. 1a). Trypsin treatment did not digest the LOA-CESH[L] band (lanes 4–5 in Fig. 1a), suggesting a failed display of LOA-CESH[L] in this system. The bands of MipAV_140_-CESH[L], InaKN-CESH[L], and InaPbN-CESH[L] in the trypsin-treated cells were appreciably thinner than those in the untreated cells (lanes 6–7, 10–11, and 12–13 in Fig. 1a). The band of YiaTR_232_-CESH[L] in trypsin-treated cells was completely disappeared, but the amount of expressed YiaTR_232_-CESH[L] protein was much less than the expressed fusion proteins in other systems (lanes 8–9 in Fig. 1a). These results indicated that the fusion proteins MipAV_140_-CESH[L], YiaTR_232_-CESH[L], InaKN-CESH[L], and InaPbN-CESH[L] were successfully displayed onto the *E. coli* cell surface with varying degrees.

After confirming that CESH[L] can be displayed on the *E. coli* cell surface, the whole-cell CESH[L] activity of each cell surface display system was examined (Fig. 1b). Surprisingly, the whole-cell activities of all cell surface display systems were very low after the induction and only the YiaTR_232_-CESH[L] system exhibited an activity of 0.34 ± 0.09 U/OD. However, after incubation at 4 °C for a few days, the activities of the InaKN-CESH[L] and InaPbN-CESH[L] systems increased significantly. Particularly, the InaPbN-CESH[L] system exhibited a very high activity of 1.67 ± 0.04 U/OD after the 2-day incubation. Combined with the trypsin accessibility assays, these results indicate that the fusion proteins with different anchoring motifs showed not only different expression and display efficiency, but also different folding levels. The InaPbN-CESH[L] system showed the highest activity after a 2-day incubation at 4 °C and was chosen for the following studies.

As a comparison, the whole-cell and lysate activity of the cells with soluble CESH[L] in an intracellular expression system (Cui et al. 2012) was 0.09 ± 0.02 U/OD and 1.29 ± 0.03 U/OD, respectively (Fig. 1c), indicating that the membrane penetrability of the substrate CES and/or the product TA is very low. Because treatments with surfactants have been used to enhance cell permeability and improve bioconversion efficiency (Rosenberg et al. 1999; Zhang and Qian 2000), the whole cells of the intracellular expression system were treated with the surfactant Triton X-100 to enhance the cell permeability. The activity of the surfactant-treated cell culture increased significantly, reaching 0.62 ± 0.05 U/OD. However, after centrifugation, the supernatant of the surfactant-treated culture retained the activity of 0.26 ± 0.03 U/OD, indicating more than one-third of activities arise from the enzyme leakage instead of the permeability for substrates/products. Furthermore, the InaPbN-CESH[L] system after 2 days of incubation at 4 °C showed an activity higher than the lysate of the intracellular expression system, suggesting an excellent expression and display level of the InaPbN-CESH[L] system without the permeability problem.

### Optimization of the InaPbN-CESH[L] surface display system

**Fig. 2:**
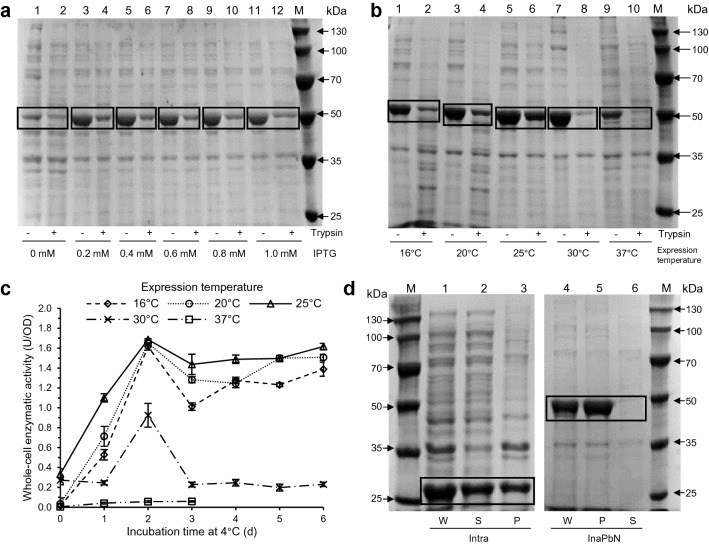
SDS-PAGE analysis of the InaPbN-CESH[L] expression. **a** The effect of the inducer IPTG concentrations. Lanes 1–12, each pair of lanes are trypsin-untreated and treated whole cells induced by IPTG with the concentrations of 0.0 mM, 0.2 mM, 0.4 mM, 0.6 mM, 0.8 mM, and 1.0 mM. Lane M, standard protein molecular weight markers. **b** The effect of the expression temperature. Lanes 1–10, each pair of lanes are trypsin-untreated and treated whole cells with the expression temperatures 16 °C, 20 °C, 25 °C, 30 °C, and 37 °C. Lane M, standard protein molecular weight markers. **c** The whole-cell enzymatic activities of InaPbN-CESH[L] with different expression temperatures. **d** Fraction analysis of intracellular expressed CESH[L] (lanes 1–3) and displayed InaPbN-CESH[L] (lanes 4–6). W, whole-cell lysate; S, supernatant after centrifugation of the cell lysate; P, pellet after centrifugation of the cell lysate. Lane M, standard protein molecular weight markers

Since the InaPbN-CESH[L] surface display system showed the highest activity among the five systems, the production conditions of this system were further optimized. To study the effect of the inducer IPTG, different concentrations of IPTG from 0.2 to 1.0 mM were used to induce the expression of InaPbN-CESH[L]. Trypsin accessibility assays showed that both the amount and the surface display efficiency of expressed InaPbN-CESH[L] were almost the same under these IPTG concentrations (Fig. 2a). The effect of cultivation temperature on InaPbN-CESH[L] expression after the induction was also studied at 16 °C, 20 °C, 25 °C, 30 °C, and 37 °C. The InaPbN-CESH[L] expression levels and the display efficiencies were different under these temperatures and the best expression temperature is 30 °C according to the SDS-PAGE analysis (Fig. 2b). However, the cells with protein expression at 30 °C showed low activity and poor stability during the incubation at 4 °C (Fig. 2c). The activity of the cells with protein expression at 16 °C, 20 °C, and 25 °C increased significantly in the first 2 days and the cells with protein expression at 25 °C showed the best activity and stability. Thus, 25 °C was used as the optimum expression temperature for the InaPbN-CESH[L] system in the following studies.

According to Fig. 2b, about half (49.6% estimated by Bio-Rad Image Lab Software) of the total InaPbN-CESH[L] proteins expressed at 25 °C were displayed on the cell surface. To check the form of undisplayed proteins, the cells expressing InaPbN-CESH[L] were lysed by ultrasonication. After centrifugation, all InaPbN-CESH[L] proteins were in the pellet and no InaPbN-CESH[L] protein was detected in supernatants, suggesting no soluble InaPbN-CESH[L] proteins were inside cells (Fig. 2d). Therefore, the un-displayed InaPbN-CESH[L] should be in inclusion bodies or buried in membranes. As a control, intracellular CESH[L] without anchoring motif was found mainly in supernatants, indicating that CESH[L] is largely soluble in *E. coli* cells with a small amount forming inclusion bodies.

### Effects of pH and temperature on enzymatic activity and stability of the InaPbN-CESH[L] system

**Fig. 3:**
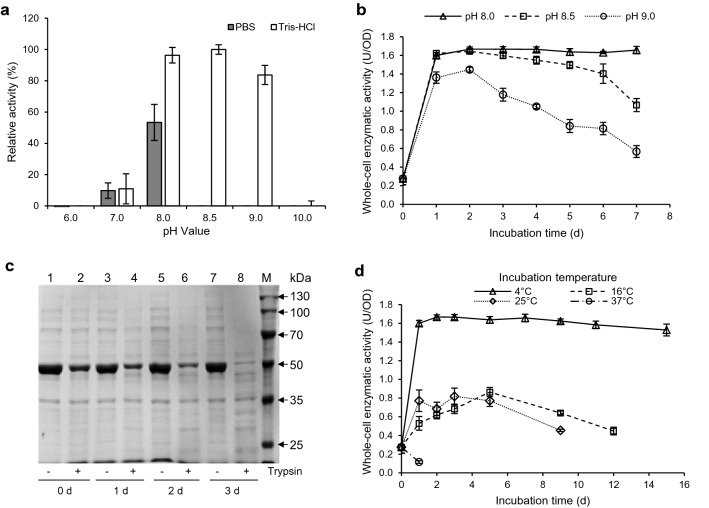
Effects of pH and temperature on surface-displayed InaPbN-CESH[L] enzymatic activity and stability. **a** Relative enzymatic activities in buffers with different pH values. The activities were measured after incubation at 4 °C for 5 h. The highest whole-cell enzymatic activity in Tris–HCl buffer (pH8.5) was set to 100%. **b** The long-term stability at 4 °C in Tris–HCl buffers with different pH values. **c** Trypsin accessibility assays of the InaPbN-CESH[L] displayed whole cells after incubation at 4 °C. Lanes 1–8, each pair of lanes are trypsin-untreated and treated whole cells with different lengths of incubation time. Lane M, standard protein molecular weight markers. **d** The long-term stability of the InaPbN-CESH[L] displayed whole cells incubated at different temperatures

Whole-cell enzymatic activities of InaPbN-CESH[L] in buffers under different pH were detected. The results showed that the InaPbN-CESH[L] surface display system had the highest enzyme activity in Tris–HCl buffer at pH 8.0 and pH 8.5 (Fig. 3a). However, the system showed higher stability at pH 8.0 than at pH 8.5 (Fig. 3b). The whole-cell enzymatic activity of the cells in Tris–HCl buffer at pH 8.0 showed no significant loss at 4 °C within 7 days. Similar to the phenomena presented in previous sections, the whole-cell enzymatic activity of the cells showed a significant increase (about five times) after one-day incubation and reached the highest value after 2-day incubation. This phenomenon suggested that there was a maturation step of the displayed InaPbN-CESH[L], which was completed in the first 2 days at 4 °C.

The increased activity observed in the InaPbN-CESH[L] system incubated at 4 °C might originate from the slow folding of displayed proteins or the slow secretion of intracellular proteins in inclusion bodies. To further address the mechanism of the phenomenon, the trypsin accessibility assays of the whole cells during the incubation were performed (Fig. 3c). The results showed that a small fraction of un-displayed proteins was continually further displayed on the cell membrane during the first three days (lanes 2, 4, 6, and 8 in Fig. 3c). Almost all InaPbN-CESH[L] proteins were displayed after 3-day incubation (lane 8 in Fig. 3c) and the total displayed proteins increased slightly during the first three days (lanes 1, 3, 5, and 7 in Fig. 3c). Considering that the activity increased about five times after one-day incubation and increased slightly since then (Fig. 3b), it could be concluded that most of the further displayed proteins in the second and third days did not significantly contribute to the activity increase during the incubation. Therefore, a maturation step, i.e., the gradual folding of the displayed proteins, occurring during the incubation at 4 °C should be the major reason for the observed activity increase.

The stability of the InaPbN-CESH[L] system incubated at different temperatures was also studied. When the cells were incubated at 37 °C, no maturation step was observed and 57.82% of whole-cell enzyme activity was lost only after one day. When incubated at 16 °C and 25 °C, the whole-cell enzyme activities rose slightly in the first few days, and thereafter, the enzyme activity began to decrease. The highest whole-cell enzyme activities were about 0.8 U/OD during the incubation. When incubated at 4 °C, the whole-cell enzyme activity reached the highest activity (1.67 ± 0.02 U/OD) after 2-day incubation. Subsequently, the activity declined slightly but maintained a constant high level. After 15-day incubation, 91.66% (1.53 ± 0.08 U/OD) of the highest activity remained (Fig. 3c). Therefore, the InaPbN-CESH[L] system is quite stable at 4 °C and suitable for long-term storage.

### Yield comparison of the surface display system and the intracellular expression system

For the intracellular expression system using the plasmid pET28a-CESH[L], the total enzymatic activity of CESH[L] in the cell lysate from 250 ml cell culture was 17,490 ± 187 U, corresponding to 55.56 ± 0.59 mg CESH[L] according to a standard curve of the activity and the purified enzyme. For the InaPbN-CESH[L] surface-display system after 2-day incubation at 4 °C, the whole-cell enzymatic activity was 1.54 ± 0.01 U for 20 μl cell culture and the total enzymatic activity was 19,193 ± 183 U for 250 ml culture, corresponding to 60.97 ± 0.58 mg active CESH[L] excluding the fusion anchoring motifs. Therefore, the InaPbN-CESH[L] surface display system displayed a higher amount of CESH[L] than the enzymes expressed in the intracellular system. Assuming that the cell number of 1.0 OD is 5 × 10^8^ per ml (Stevenson et al. 2016), the display level of the InaPbN-CESH[L] system was estimated to be about 1.9 × 10^6^ molecules per cell. The final cell density of the intracellular expression system using the plasmid pET28a-CESH[L] was slightly lower than the InaPbN-CESH[L] surface-display system (OD_600_ 4.5 vs. 5.0), and the CESH[L] molecular number of the intracellular expression system was estimated to be 1.9 × 10^6^, indicating the similar amount of CESH[L] molecules were produced in each cell of both systems.

### Evaluation of the reusability of the InaPbN-CESH[L] display system

**Fig. 4:**
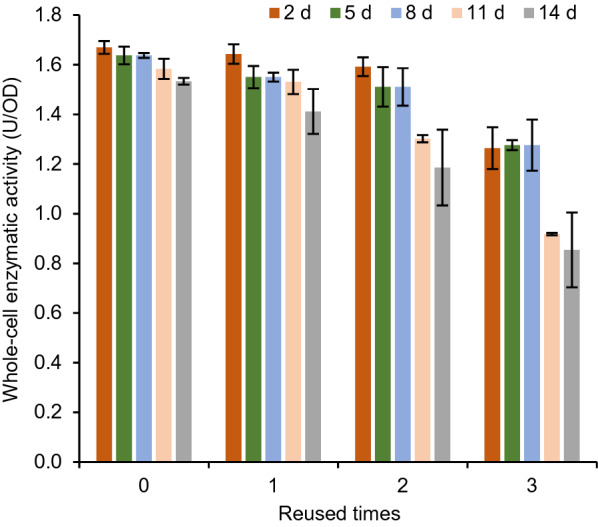
Repeated use of the InaPbN-CESH[L] surface display system. The cells after protein expression were incubated at 4 °C for different days and then the whole-cell enzyme activities were repetitively assayed four times. After each enzymatic reaction, the cells were centrifuged and washed with the assay buffer, and then the whole-cell enzyme activities were immediately assayed again as repetitive usage

After incubation of the InaPbN-CESH[L] display cells at 4 °C for different days, three rounds of whole-cell enzymatic activity measurements were performed to evaluate the reusability of the system. For the cells with 2-day incubation, three usages of the whole cells remained about 98.44%, 95.39%, and 75.73% of their original activity. Furthermore, the cells with 5- and 8-day incubation also showed good reusability similar to the cells with 2-day incubation. For the incubation time longer than eight days, the reusability of whole cells was impaired, but the system with 14-day incubation still showed about 51.17% of the highest activity in the third usage of the cells (Fig. 4). These results indicated that the InaPbN-CESH[L] display system has good reusability.

## Discussion

In this research, five anchoring motifs were screened to construct the CESH[L] surface display system and the results showed that the N-terminal INP truncation InaPbN is the most suitable anchoring motif for CESH[L] surface display. To our knowledge, this is the first report on the CESH[L] surface display system that overcomes the membrane permeability problem of intracellular CESH[L] whole-cell biocatalysts. The results in this research showed that different anchoring motifs have significantly different efficiencies for CESH[L] surface display and two kinds of INP-mediated systems achieved higher surface display levels than other systems. Even for the two INP truncation motifs, the surface display efficiency, the whole-cell enzymatic activity, and the stability are also different. Therefore, to obtain an efficient surface display system for a specific protein, various anchoring motifs should be screened and the most suitable anchoring motif may highly depend on the passenger protein. The screening protocol used in this study is valuable for whole-cell biocatalyst construction for other industrial enzymes.

An interesting discovery in this study is the maturation step of the displayed InaPbN-CESH[L], which was rarely reported in other enzyme display systems. Two surface display systems using an INP N-terminal domain as the anchoring motif for triphenylmethane reductase and carboxylesterase have been reported to have a significant increase in the activity (to 137% and 280%) after incubation at 4 °C for eight and twelve days, respectively (Ding et al. 2020; Gao et al. 2014), but the reason for the activity increase was not investigated. Most other studies using the INP N-terminal domain as the anchoring motif did not observe the maturation step, suggesting that the maturation process mainly depends on the client protein. The existence of the maturation step suggests that the folding of the secreted CESH[L] is quite slow and cannot be completed immediately after its location on the cell surface. The slow folding of CESH[L] is consistent with the observation that part proteins of intracellular CESH[L] formed inclusion bodies (Fig. 2d). The surface display of CESH[L] can prevent their aggregation and thus restore their activities after a long period. This demonstrated the advantages of a surface display system for proteins with low stability. The maturation step discovered in this study should also be considered in the development of cell surface display systems for other industrial enzymes.

Total enzymatic activities of the InaPbN-CESH[L] surface display system are higher than that of the intracellular expression system, giving a high display level of 1.9 × 10^6^ molecules per cell. In previous studies, the number of displayed protein molecules using OmpA-derived anchoring motif is in the range of 1.5 × 10^4^ to 1.8 × 10^5^ per cell, limited by the number of bearable autotransporters on the cell membrane (Jose and Meyer 2007; Quehl et al. 2017; Smith et al. 2015). Our results suggest that the INP-based anchoring motif might display one order more molecules on *E. coli* cells, in agreement with that INP-derived anchoring motif has little interference on cell viability (van Bloois et al. 2011).

The results in this study demonstrate many benefits of using the surface display system. The InaPbN-CESH[L] system not only has a higher total activity than the intracellular expression system, but also can be directly used as whole-cell catalysts without the interference of the low membrane permeability and the loss of enzymes during the purification process of intracellular CESH[L]. The high whole-cell enzymatic activity of InaPbN-CESH[L] can remain as long as 15 days at 4 °C, which is consistent with previously reported INP-mediated surface display systems (Gao et al. 2014; Song et al. 2015). Moreover, the surface-displayed CESH[L]s can be reused several times without much activity loss, which is valuable in industrial applications. All these advantages indicate that the InaPbN-CESH[L] surface display system provides a convenient, highly efficient, and valuable method for industrial L(+)-TA production.

## Conclusions

In this study, the efficient whole-cell biocatalyst InaPbN-CESH[L] was obtained for bacterial cis-epoxysuccinic acid hydrolase by screening five different surface display systems. The whole-cell biocatalyst overcomes the problems of low stability of purified CESH[L] and the low cell-permeability of whole-cell catalysts with intracellular CESH[L]. Furthermore, the optimized total activity of the InaPbN-CESH[L] system is higher than the total lysate activity of the intracellular CESH[L] overexpression system, indicating a very high CESH[L] display level. The whole-cell biocatalyst exhibited good storage stability and considerable reusability and provides a valuable system for industrial L(+)-tartaric acid production.

## Data Availability

The datasets used during the current study are available from the corresponding author on reasonable request.
